# Experimental Passage of St. Louis Encephalitis Virus *In Vivo* in Mosquitoes and Chickens Reveals Evolutionarily Significant Virus Characteristics

**DOI:** 10.1371/journal.pone.0007876

**Published:** 2009-11-17

**Authors:** Alexander T. Ciota, Yongqing Jia, Anne F. Payne, Greta Jerzak, Lauren J. Davis, David S.Young, Dylan Ehrbar, Laura D. Kramer

**Affiliations:** 1 The Arbovirus Laboratories, Wadsworth Center, New York State Department of Health, Slingerlands, New York, United States of America; 2 School of Public Health, State University of New York at Albany, Albany, New York, United States of America; Institut Pasteur, France

## Abstract

St. Louis encephalitis virus (SLEV; *Flaviviridae*, *flavivirus*) was the major cause of epidemic flaviviral encephalitis in the U.S. prior to the introduction of West Nile virus (WNV) in 1999. However, outbreaks of SLEV have been significantly more limited then WNV in terms of levels of activity and geographic dispersal. One possible explanation for these variable levels of activity is that differences in the potential for each virus to adapt to its host cycle exist. The need for arboviruses to replicate in disparate hosts is thought to result in constraints on both evolution and host-specific adaptation. If cycling is the cause of genetic stability observed in nature and arboviruses lack host specialization, then sequential passage should result in both the accumulation of mutations and specialized viruses better suited for replication in that host. Previous studies suggest that WNV and SLEV differ in capacity for both genetic change and host specialization, and in the costs each accrues from specializing. In an attempt to clarify how selective pressures contribute to epidemiological patterns of WNV and SLEV, we evaluated mutant spectra size, consensus genetic change, and phenotypic changes for SLEV *in vivo* following 20 sequential passages via inoculation in either *Culex pipiens* mosquitoes or chickens. Results demonstrate that the capacity for genetic change is large for SLEV and that the size of the mutant spectrum is host-dependent using our passage methodology. Despite this, a general lack of consensus change resulted from passage in either host, a result that contrasts with the idea that constraints on evolution in nature result from host cycling alone. Results also suggest that a high level of adaptation to both hosts already exists, despite host cycling. A strain significantly more infectious in chickens did emerge from one lineage of chicken passage, yet other lineages and all mosquito passage strains did not display measurable host-specific fitness gains. In addition, increased infectivity in chickens did not decrease infectivity in mosquitoes, which further contrasts the concept of fitness trade-offs for arboviruses.

## Introduction

*St. Louis encephalitis virus* (SLEV) is a member of the genus *flavivirus*, family *Flaviviridae*. SLEV is a close relative of *West Nile virus* (WNV) and other members of the Japanese encephalitis serocomplex [1]. Like WNV, SLEV is predominantly maintained in a transmission cycle between ornithophilic mosquitoes and birds. The dominant vectors of both SLEV and WNV in N. America are mosquitoes in the *Culex* genus [2], [3]. SLEV was the major cause of epidemic flaviviral encephalitis in the United States prior to the introduction of WNV into North America. More than 4,600 human infections were reported between 1964 and 2005 [4]. However, since its emergence in the United States in 1999, WNV has spread to 48 states and caused illness in more than 20,000 humans (http://www.cdc.gov/ncidod/dvbid/westnile/surv and control.htm). Although SLEV has occasionally been transported between geographic regions both within and outside the U.S. [5], [6], phylogenetic analyses indicate that SLEV is predominantly maintained locally, with strains generally clustering according to geographic origins [7]. Understanding the more contained nature of SLEV activity relative to that of the widespread dissemination of WNV could be potentially important in determining the factors which are significant in dictating the breadth of arbovirus activity in general.

The need for arboviruses to replicate in disparate hosts is often thought to result in constraints on both evolution and host-specific adaptation. This hypothesis is consistent with the fact that the magnitude of genetic change observed with arboviruses in nature has generally not been consistent with the enormous potential for change inherent to RNA viruses [8]. If differing selective pressures resulting from cycling are the cause of genetic stability observed in nature, then sequential passage in a single host species should result in the accumulation of mutations which otherwise would be purged. Studies done previously with WNV demonstrate that significant intrahost genetic diversity is generated with both sequential and alternate *in vivo* passage; and the source of this genetic diversity is host-specific due to more relaxed purifying selection in mosquitoes [9], [10]. These studies, which also show no difference in the number of mutations fixed in alternate and sequentially passaged populations, do not necessarily support the idea of a dampened rate of genetic change as a consequence of host cycling. Here, in an attempt to begin to clarify the role of selective pressures in the differing epidemiological patterns of WNV and SLEV, we evaluated both host-specific mutant spectra size and consensus genetic change for SLEV following sequential passage in either *Culex pipiens* Linneaus mosquitoes or chickens. Previous studies in mosquito cell culture suggest SLEV may produce and maintain much more limited intrahost mutant spectra relative to WNV during sequential passage [11]. Differing levels of genetic diversity in nature could contribute to differences in adaptability of virus populations and, consequently, differences in host and geographic range, as well as in overcoming seasonal bottlenecks. Beyond this, minority sequences have been clearly implicated in contributing to other phenotypes including both viral fitness and viral pathogenesis [9], [12]–[14]. Evolutionary theory also would predict that arboviruses need to be generalists in order to replicate in vastly different environments, and that the cost of this generalism would be suboptimal adaptation to each individual host [15]. If this were the case, sequential passage in a single host as completed here also should result in a more specialized virus which is better suited for replication in that host. Previous studies with both alpahviruses [16], [17]–[19] and flaviviruses [11], [20] have demonstrated host specialization with passage, yet previous passage of SLEV in *Cx. pipiens* demonstrated a lack of adaptation in *Cx. pipiens* [21]. In those studies 10 plaque forming units (pfu) of secreted virus from a single day was used for passaging. Here, in order to (a) allow for direct comparison to previous WNV studies assessing mutant spectra sizes [9], and (b) determine if a larger, more diverse population passage enabled further adaptation, we passaged 100 pfu of virus from the entire mosquito. Similar *in vivo* passage of WNV demonstrated that further adaptation to *Cx. pipiens* mosquitoes was attainable, yet no measurable cost resulted in avian hosts in terms of levels and rates of viremia production. These results, together with previous *in vitro* studies suggest that even with host specialization, WNV may retain its status as a generalist [11]. This idea of the absence of a significant fitness trade-off has also been demonstrated in studies with vesicular stomatitis virus (VSV) [22], [23]. On the other hand, *in vitro* results with SLEV do demonstrate a cost in some hosts as a result of specialization [11]. By continuing to assess these characteristics *in vivo* we can begin to determine the genetic correlates of host range and specialization and, therefore, shed light on understanding factors that influence variation in arbovirus evolution and activity in nature.

## Methods

### Ethics Statement

All animal use was approved by the Wadsworth Center Institution of Animal Care and Use Committee (06-355).

### Experimental Hosts

*Cx. pipiens* egg rafts were collected in Pennsylvania in 2004 and colonized at the Wadsworth Center insectary facilities. Mosquito rearing and preparation for experimentation were carried out as previously described [9], [11]. White leghorn chickens (*Gallus gallus*) used for experimental passage were received from Charles River breeding labs (Boston, MA). Pathogen-free chicken eggs were obtained from Sunrise Farms (Catskill, NY), hatched in an incubator (G.Q.C) at the Arbovirus Laboratories, Wadsworth Center, and used for experimentation following hatching. Chickens were housed in metal cages with individual light sources and daily fresh food, water, and resting pads.

### Viruses

A biological clone of SLEV strain Kern (217.3.1.1) was used for commencement of passage studies and as a control (SLEV P0) in subsequent experimentation. The clone was isolated by three rounds of plaque purification on Vero cells and was derived from the SLEV Kern 217 isolated in 1989 from *Culex tarsalis* from Kern County, CA (obtained from Dr. William Reisen, University of California at Davis;[7]. It has previously been demonstrated that growth kinetics of SLEV P0 and the wildtype SLEV Kern 217 are similar in all hosts (data not shown). Virus strains derived from passage are denoted as either mosquito-passaged (SLEV MP) or chicken-passaged (SLEV CP) together with the number of passages completed.

### Experimental Passage

**Figure 1:**
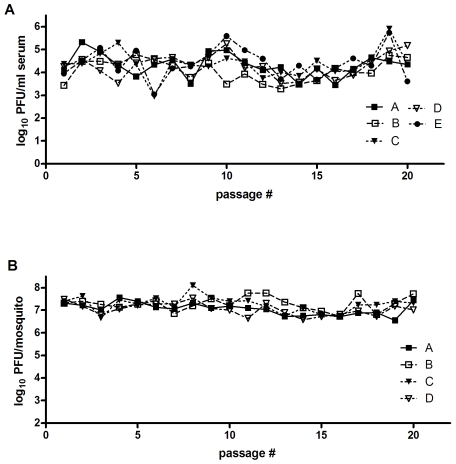
Viral load of individual lineages of SLEV during sequential passage in chickens (A) or mosquitoes (B).

Sequential passages were carried out 20 times in either chickens or *Cx. pipiens* mosquitoes using SLEV P0. Four (mosquito) or five (chicken) separate lineages were maintained throughout passage (A–E). Passage in mosquitoes was carried out by intrathoracic inoculation of 0.1 ul as previously described [9], [24]. Four female mosquitoes, 4–7 days old, were inoculated with 100 pfu for each lineage at every passage. At 7 days post inoculation (p.i.) whole mosquitoes were placed into 2.0 ml microcentrifuge tubes with 1 ml mosquito diluent (MD; 20% heat-inactivated fetal bovine serum [FBS] in Dulbecco's phosphate-buffered saline plus 50 µg/ml penicillin/streptomycin, 50 µg/ml gentamicin, and 2.5 µg/ml Fungizone) plus one 5 mm BB (Daisy, Rogers, Arkansas). Samples were homogenized for 30 seconds at 20 Hz in a Mixer Mill MM301 (Retsch, Haan, Germany). Debris was then pelleted by centrifugation at 10,000 rpm for 5 minutes and titrated by plaque assay in duplicate on Vero cells as previously described [25]. The highest titer sample from each lineage was diluted and used for subsequent passage (Fig. 1).

Passage in chickens was carried out by subcutaneous (s.c.) inoculation of two 5-day old chickens per lineage with 100 ul of virus passage as previously described [21]. At two days p.i., animals were anesthetized using 100 ul Sleepaway (Fort Dodge Animal Health, Fort Doge, IA) and blood collected by cardiac puncture using 3 ml serum separator tubes (Fisher healthcare, Houston, TX) after which chickens were euthanized. Serum was obtained by spinning whole blood at 4500 r.c.f. for 15 min, and subsequently titrated by plaque assay in duplicate on Vero cells. The highest titer sample from each lineage was appropriately diluted and used for subsequent passage (Fig. 1).

### Viral Growth Kinetics and Infectivity in Mosquitoes

Female *Cx. pipiens* were infected by intrathoracic inoculation for both determination of infectious dose-50 (ID_50_; dose at which 50% of experimental hosts become infected) and growth of individual virus strains. The ID_50_ for each SLEV strain was determined by inoculation of 10–25 mosquitoes per dilution using ten-fold increasing concentrations of virus from 0.1 pfu, and screening for infection by plaque assay on Vero cell culture at 7 days p.i. Calculations of ID_50_ were done using the Reed-Muench formula. Inoculations for mosquito growth kinetics were done with 100 pfu and viral titer was determined for 8–10 mosquitoes/timepoint. Mosquitoes for both assays were collected and treated as previously described for experimental passage [21].

### Viremia Kinetics and Infectivity in Chickens

One-day old chickens hatched on-site were used for experimentation following passage. For viremia determination, chickens were inoculated s.c. with 10 pfu of virus and bled from the brachial vein on days 1–5 p.i. as previously described [21]. Whole blood was processed as described in passage methodology and viral titers were used to generate viremia curves. For ID_50_ experiments, 5–6 chickens/dose were inoculated s.c. with 10, 1.0, 0.1, or 0.01 pfu. At day 14 p.i., chickens were bled by cardiac puncture, after which chickens were euthanized, and serum was obtained. Serum was tested for the presence of WNV-specific antibody using the plaque reduction neutralization test (PRNT) as previously described [26], [27]. The proportion of infected chickens for each dose was determined and calculations of ID_50_ were done using the Reed-Muench formula.

### Molecular Cloning and Population Analysis

Production and analysis of clones was performed basically as previously described [12], [28]. RNA was extracted from infected specimens with Qia-amp viral RNA extraction kit (Qiagen, Valencia, CA) and RT-PCR was conducted using primers designed to amplify the 3′ 1302 nt of the SLEV envelope (E) coding region and the 5′ 3325 nt of the SLEV non-structural protein 1 (NS1) coding region. RT of 5 µl RNA was performed with Sensiscript RT (Qiagen) at 45°C for 40 min. RT reactions were followed by heat inactivation at 95°C for 5 min. The resulting cDNA was used as a template for PCR amplification. SLEV cDNA was then amplified with a ‘high-fidelity’ protocol using *Pfu*Ultra (published error rate  = 4.3×10^−7^; Stratagene, La Jolla, CA), according to the manufacturer's specifications. Amplification was carried out for 40 cycles at 94°C for 30 sec, 50°C for 30 sec and 72°C for 4 min, and one cycle at 72°C for 10 min. PCR products were visualized on a 1.5% agarose gel and DNA was recovered by using a MinElute Gel Extraction kit (Qiagen) as specified by the manufacturer. The recovered DNA was ligated into the cloning vector pCR-Blunt II-TOPO (Invitrogen, Carlsbad, CA) and transformed into One Shot TOP10 Electrocomp *E.coli* cells according to the manufacturer's protocol. Kanamycin resistance was used for initial detection of transformed colonies. Colonies were then screened by direct PCR using primers specific for the desired insert. Plasmid DNA was purified by using a QIAprep Spin Miniprep kit (Qiagen) as specified by the manufacturer. Sequencing was carried out by using five pairs of overlapping SLEV primers together with T7 and SP6 primers. Sequencing was performed at the Wadsworth Center Molecular Genetics Core using ABI 3700 and 3100 automated sequencers (Applied Biosystems, Foster City, CA). Fifteen to twenty-four clones per sample were sequenced. Sequences were compiled, edited, and aligned using DNASTAR software package (Madison, WI). Individual clones were compared to consensus sequences as previously described [12]. The percentage of nucleotide mutations (total number of mutations divided by total number of bases sequenced), amino acid mutations (total number of amino acid changes divided by total number of amino acids sequenced), and the sequence diversity (percent of clones with at least one difference from consensus) were used as indicators of genetic diversity. Normalized Shannon entropy (S_n_) was calculated based on frequency of genotypes in populations as follows: Shannon entropy (S_n_) = ∑-_i_ P_i_ lnP_i_/ln N, where P_i_  =  frequency of individual genotype and N  =  number of clones sequenced. Sn values range from 0 (completely homogeneous) to 1(completely heterogeneous)_._

### Full-Genome Sequencing

RNA was extracted from SLEV using RNeasy (Qiagen, Valencia, CA) according to manufacturer's protocol and sequencing was carried out as previously described [29]. One-step RT-PCR (Qiagen) was conducted using primers to generate nine overlapping PCR products. Reverse transcription reactions were carried out at 50°C for 30 min, followed by inactivation of the transcriptase at 95°C for 15 min. Amplification was then carried out for 40 cycles at 94°C for 20 sec, 55°C for 30 sec, 72°C for 2 min, with final elongation at 72°C for 10 min. PCR products were visualized on a 1.5% gel and then bands were then allowed to run through 1% Nusieve GTG low-melting agarose (Cambrex BioScience, Rockland, ME). Sequencing was performed with ABI 3700 automated sequencers (Applied Biosystems, Foster City, CA) using overlapping primers with a minimum of two-fold redundancy. Sequences were compiled and edited by using DNASTAR software package (Madison, WI).

## Results

### Intrahost Genetic Diversity during *In Vivo* Passage

**Figure 2:**
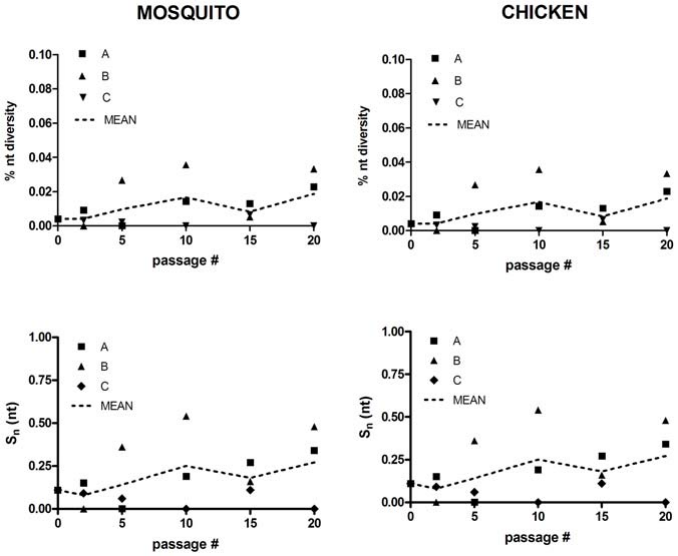
Intrahost nucleotide diversity of bases 1315–3325 for individual lineages of SLEV during sequential passage in chickens or mosquitoes. Nt diversity is equivalent to the total # of mutations relative to the consensus/total # of bases sequenced. S_n_(nt) refers to normalized Shannon entropy based on the frequency of individual genotypes.

**Figure 3:**
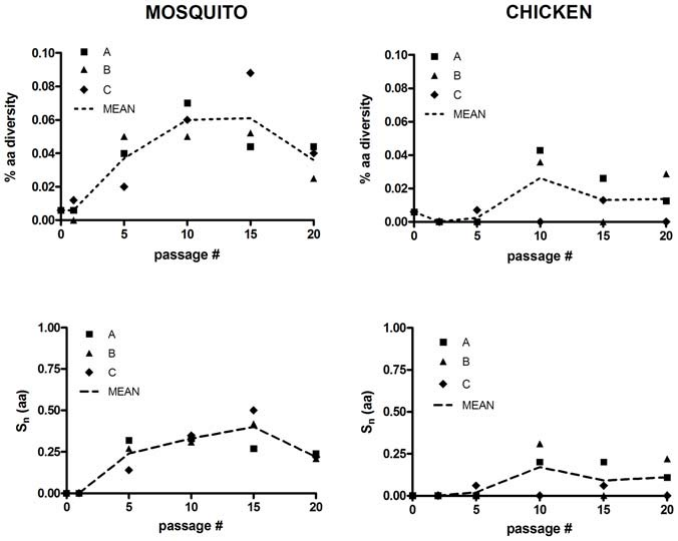
Intrahost amino acid diversity for individual lineages of SLEV during sequential passage in chickens or mosquitoes. AA diversity is equivalent to the total # of amino acid substitutions relative to the consensus/total # sequenced. S_n_(aa) refers to normalized Shannon entropy based on the frequency of individual amino acid genotypes.

Intrahost genetic diversity of SLEV was determined before (SLEV P0) and after 0, 1(2), 5, 10, 15, and 20 passages in *Cx. pipiens* mosquitoes (MP) or chickens (CP) in order to assess fluctuations in the size of the mutant spectrum accrued during sequential *in vivo* passage. Three lineages (A, B, and C) were evaluated for both MP and CP strains to provide an accurate representation of the size of the host-specific mutant spectra. Intrahost diversity of chicken-passaged lineage D (CP20D) also was evaluated following identification of unique phenotypic changes. Genetic diversity, i.e., nt and aa variation, was generally higher during passage in mosquitoes than during passage in chickens (Figs. 2 and 3). Mutant spectra size expanded for 10 (lineage A and B) or 15 (C) mosquito passages, followed by subsequent purification of the population by passage 15 or 20, respectively (Fig. 2). Amino acid diversity demonstrated the same general trend as nt diversity and much of the variation among lineages was reduced when aa diversity is evaluated on the level of sequence variation (aa S_n_; Fig. 3). Following 20 passages approximately 20.0% of sequences analyzed possessed at least one aa change relative to the consensus sequence in the region analyzed. The peak nt diversity (0.066%), nt sequence entropy (0.72), aa diversity (0.088%), and aa sequence entropy (0.50) for mosquito-passaged strains was measured with lineage C at passage 15. This level of genetic diversity is equivalent to an average of seven nt changes relative to the consensus sequence per SLEV genome (11 kb), with three of these changes being nonsynonomous.

Mutant spectra size during passage of SLEV in chickens was highly variable among lineages using all measures of diversity (Figs. 2 and 3). Lineage C remained highly homogeneous throughout passage, while lineages A and B displayed fluctuations from passage 2 to passage 20. Mean nt diversity peaked at passage 20 (0.021%) despite a lack of variation in lineage C. The peak nt diversity(0.036%) and nt sequence entropy (0.55) for chicken-passaged SLEV was measured in lineage B after 10 passages, yet the peak aa diversity (0.047%) and aa sequence entropy (0.34) was observed in passage 20 of lineage D.

### Consensus Sequencing

Full-genome sequences of SLEV before (P0) and after (MP20A, MP20B, CP20A, CP20B, and CP20D) passage were generated and compared in order to determine the extent of fixed, consensus change which occurred as a result of passage in each host. Although variation between lineages existed, overall consensus level change following passage was limited (table 1). For both SLEV MP20B and CP20B, in fact, other than a mixed population identified at a single site (A8057R and C2025Y, respectively), no consensus change was identified. In addition, just a single substitution was identified in SLEV CP20A (G2845A), resulting in highly conservative arginine to lysine amino acid substitution in the NS1 gene. SLEV MP20A and CP20D were slightly more variable with 6 and 4 nt substitutions respectively, resulting in 4 (MP20A) and 2 (CP20D) amino acid changes. In addition, a mixed population was identified at a single position in MP20A (A5695R). All amino acid changes were fairly conservative, with the exception of the A6981G nt substitution which resulted in arginine (charged polar) to glycine (nonpolar) amino acid change in the NS4B gene. Surprisingly, this change was identified in both MP20A and CP20D.

**Table 1:** Consensus sequence changes of SLEV following passage in chickens (CP) or *Cx. pipiens* mosquitoes (MP).

Strain	nt change	aa change	location
***SLEV MP20A***	*G1635A*	*none*	*ENV*
	*G1663A*	*R552H*	*ENV*
	*A3107G*	*none*	*NS1*
	*T5659A*	*F1854Y*	*NS3*
	*A5695R(A/G)*	*K1866R*	*NS3*
	*C6583T*	*A2162V*	*NS4A*
	*A6981G*	*R2295G*	*NS4B*
***SLEV MP20B***	*A8057R(A/G)*	*none*	*NS5*
***SLEV CP20A***	*G2845A*	*R916K*	*NS1*
***SLEV CP20B***	*C2025Y(T/C)*	*none*	*ENV*
***SLEV CP20D***	*C866T*	*none*	*M*
	*A6981G*	*R2295G*	*NS4B*
	*A7971G*	*K2625Q*	*NS5*
	*G8084A*	*none*	*NS5*

### Viral Growth Following Vertebrate Passage

SLEV titers during passage were variable among lineages and passage number, but overall did not display any trend toward increasing or decreasing titer as a result of passage (Fig. 1). Changes in plaque morphology on Vero cell culture were observed, with a general increase in plaque size noted for all lineages, most notably in SLEV CP20D (data not shown).

**Figure 4:**
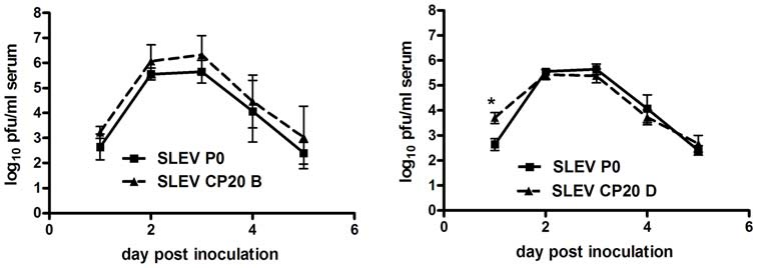
SLEV viremia kinetics in 1-day old chickens before (P0) and after (CP20) passage in chickens. Data points represent means of 4–5 chickens +/− S.D. Statistically significance differences are indicated by an asterisk (t-test, p<0.05).

In order to quantify changes in viral growth kinetics in chickens following passage, viremia levels were measured on days 1–5 p.i. of SLEV before (P0) and after (CP20B and D) passage in chickens. Viral titers of inputs for chicken viremia experiments were approximately 10 pfu for all groups, and did not significantly differ from each other. Results demonstrate that, overall, growth kinetics were similar before and after passage (Fig. 4). For CP20B, no statistical difference was measured at any timepoint relative to SLEV P0 (t-test, p>0.05). Despite this, at all timepoints p.i., mean viremia levels were higher for CP20B and some uncharacteristically high viremia levels were measured in individual chickens infected with this strain. For instance, at days 2 and 3 p.i. viremia levels of 6.90 and 7.30 log_10_ pfu/ml, respectively, were measured in a single chicken infected with CP20B. The highest individual levels of viremia measured on days 2 and 3 p.i. for a chicken infected with SLEV P0 were 5.81 and 6.45 log_10_ pfu/ml, respectively. No individual chickens infected with CP20D demonstrated such high levels of viremia on days 2 and 3 p.i., yet significantly elevated day 1 viremia levels were measured in this group relative to SLEV P0 (3.70 vs. 2.64 log_10_ pfu/ml; t-test, p = 0.01). Beyond day 1 p.i. viremia levels were similar for SLEV CP20D and SLEV P0.

### Viral Growth Following Invertebrate Passage

SLEV titers were comparable throughout passage and among lineages, with no indication of increasing or decreasing viral load as a result of passage (Fig. 1). Modest alterations in plaque morphology on Vero cell culture were noted with some increases in plaque heterogenity resulting from the appearance of some smaller plaques in all lineages. On average, alterations in plaque morphology were much more pronounced in chicken-passaged SLEV (data not shown).

**Figure 5:**
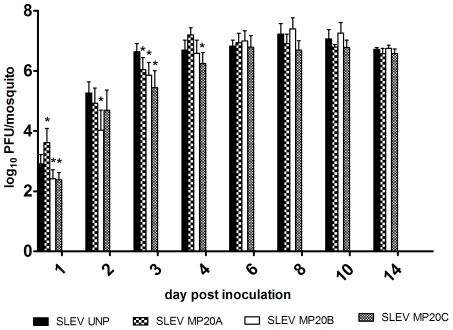
SLEV growth kinetics in *Cx. pipiens* mosquitoes before (P0) and after (MP20) passage in *Cx. pipiens* mosquitoes. Data points represent means of 8–10 mosquitoes +/− S.D. Statistically significance differences are indicated by an asterisk (t-test, p<0.05).

Viral titers were determined following IT inoculation of *Cx. pipiens* before (P0) and after (MP) 20 passages in *Cx. pipiens* in order to evaluate the extent to which mosquito passage altered SLEV growth kinetics. Experiments were done in parallel and input titers for each strain tested were equivalent (100 pfu). Overall, growth kinetics were generally similar before and after passage, indicating no substantial change in replicative ability as a result of passage (Fig. 5). Despite this, significant differences between P0 and MP virus strains were measured at some individual timepoints (Fig. 5; t-test, p<0.05). Of these, just one MP strain on a single day showed a significantly higher viral titer than SLEV P0 (SLEV MP20A day 1; p<0.05). All other differences measured between SLEV P0 and MP strains indicated significantly lower titers for MP relative to P0 (days 1–4 p.i.; p<0.05). Taken together, these results demonstrate that on average initial rate of growth is lower for MP strains, yet the inconsistency between strains and timepoints calls into question the biological significance of these differences. Beyond day 6 p.i. all titers are statistically similar.

### Virus Infectivity *In Vivo*

In order to assess possible changes in virus infectivity resulting from passage, infection rate at various doses and ID_50_ of SLEV were determined in chickens or mosquitoes before and after passage (tables 2 and 3). A single lineage of chicken-passaged SLEV (CP20D) was selected for infectivity testing in chickens as a result of the elevated day 1 p.i. viremia levels relative to SLEV P0 (Fig. 4). Results indicate significantly higher proportions of chickens infected at 1.0 and 0.1 pfu for SLEV CP20D relative to SLEV P0 (Chi-squared, p<0.05). The 0.01 pfu dose of SLEV P0 was not evaluated in chickens due to the fact that no infection occurred at the next higher dose (0.1 pfu), yet two of six chickens infected with 0.01 pfu of SLEV CP20D did become infected (33.3%; table 2). Calculation of ID_50_ from these data indicated SLEV CP20D was approximately 30-fold more infectious than its parental strain (P0) prior to chicken passage (0.03 pfu v. 0.96 pfu; table 2). In order to determine if increased infectivity in chickens resulted in a compromised ability to infect mosquitoes, infectivity of SLEV CP20D was also assessed in *Cx. pipiens* mosquitoes. Results indicated no significant differences in infection rates at any dose (Chi-squared, p>0.05) and equivalent ID_50_ values for SLEV before and after chicken passage (table 3). Similar to growth kinetics results, mosquito-passaged SLEV (MP20A and MP20B) also showed no significant change in *Cx. pipiens* infectivity as a result of passage (table 3). Although infection rates were slightly higher for SLEV MP20A or B relative to SLEV P0 at some doses, no differences were statistically different (Chi-squared, p>0.05) and ID_50_ values were equivalent (table 3).

**Table 2:** Proportion of chickens infected following inoculation of 10-fold dilutions of SLEV before (P0) and after (CP) passage in chickens.

Strain	10.0 pfu	1.0 pfu	0.1 pfu	0.01 pfu	ID_50_ 1
*SLEV PO*	*6/6 (100%)*	*3/6 (50%)*	*0/6 (0%)*	*-*	*0.96*
*SLEV CP20D*	*6/6 (100%)*	*6/6 (100%)* *	*4/6 (67%)* *	*2/6 (33%)*	*0.03*

*Chi-squared, p<0.05.

1Calculated using Reed-Muench formula.

**Table 3:** Proportion of *Cx. pipiens* mosquitoes infected following inoculation of 10-fold dilutions of SLEV before (P0) or after passage in mosquitoes (MP) or chickens (CP).

Strain	10.0 pfu	1.0 pfu	0.1 pfu	0.01 pfu	ID_50_ 1
*SLEV PO*	*15/15 (100%)*	*13/15 (87%)*	*18/25 (72%)*	*0/10 (0%)*	*0.05*
*SLEV MP20A*	*15/15 (100%)*	*15/15 (100%)*	*14/21 (67%)*	*1/7 (14%)*	*0.05*
*SLEV MP20B*	*15/15 (100%)*	*13/15 (87%)*	*16/21 (76%)*	*1/7 (14%)*	*0.05*
*SLEV CP20D*	*15/15 (100%)*	*13/15 (87%)*	*14/20 (70%)*	*1/10 (10%)*	*0.05*

1Calculated using Reed-Muench formula.

## Discussion

SLEV is maintained in nature by cycling between *Culex* species mosquitoes and avian hosts. Although it has been successfully established in the U.S. since its isolation in 1933, SLEV has never reached levels of activity observed in recent years with WNV despite the genetic and ecological similarities between these two viruses (www.cdc.gov/ncidod/dvbid; [30]). In fact, it has been suggested that SLEV has been displaced by WNV from a number of locations [31]. It is hypothesized that the need for arboviruses to replicate in divergent vertebrate and invertebrate hosts has constrained both evolution and host-specific adaptation yet, despite numerous studies testing this hypothesis, the specifics of the selective pressures and evolutionary trade-offs which shape arbovirus evolution are still poorly understood. Much of this knowledge gap stems from the fact that virus-specific differences clearly exist; and attempting to apply universal explanations for arbovirus evolution based on experimental results has proven difficult [11], [16], [18], [20], [22]. Beyond this, the majority of the experimental evolution studies have been done in cell culture systems which are generally not accurate models of natural host systems. *In vitro* studies are highly useful tools in establishing and testing hypotheses, yet it is *in vivo* studies that will ultimately provide the necessary data to advance our understanding of arbovirus evolution in nature. Other than limited studies with Ross River virus [32], Venezuelan equine encephalitis virus (VEEV; [19], and WNV [9], [10], [21], *in vivo* evaluation of arbovirus evolution in the laboratory is still lacking. Here, in an effort to expand on these limited studies we evaluated genetic and phenotypic changes of SLEV resulting from sequential passage in *Cx. pipiens* mosquitoes or chickens. If host cycling does in fact restrict genetic change and host-specific adaptation as hypothesized then sequential passage as completed here should result in significant accumulation of mutations and measurable gains in host-specific fitness. Virus titers during passage in mosquitoes or chickens did not indicate any substantial gains in viral load at the time of harvest (Fig. 1). Although there were fluctuations in viral titers over time and among lineages for chicken-passaged SLEV, there was no consistent trend observed. In addition, viral growth kinetics following passage in general was not consistent with any substantial gains in replicative fitness (Figs. 3 and 4). It is possible that direct competition assays may have revealed more subtle differences in viral fitness, yet previous *in vitro* studies suggest that individual growth kinetics are a sufficient screen for identifying significant gains in replicative fitness [11]. In chickens, although significantly higher viremia levels were not measured on any day p.i. for the SLEV CP20B infected chickens as a group, it is notable that some individual chickens did in fact display viremia titers which were more elevated than levels typically observed for SLEV replicating in chickens (Fig. 4). Variation among viral titers in chickens is normal following infection with SLEV, yet the fact that the highest levels were seen in the SLEV CP20B group exclusively suggests more adapted genetic variants may have emerged in those particular chickens. Although a lack of consensus change was observed for SLEV CP20B (table 1), this strain did possess a substantial mutant spectrum at passage 20 and it is therefore feasible that some minority variants were more suited for avian replication (Fig. 2). For SLEV CP20D, significantly higher viremia was measured on day 1 p.i. only (Fig. 4). Infectious dose experiments confirmed that this difference could be attributed to a greater than 30-fold increase in infectivity resulting from chicken passage (table 2). These results, together with the observation that plaque size on mammalian cell culture was on average larger following chicken passage, suggest that although SLEV is already highly adapted to its avian host, some further vertebrate-specific adaptation is certainly attainable.

The finding of equivalent mosquito infectivity for CP20D (table 3) demonstrates that such adaptation does not necessarily come at a cost in the alternate host as would be predicted by evolutionary theory [15]. This lack of fitness trade-off has been observed previously with VSV *in vitro* [22], [23], SLEV and WNV *in vitro* [29] and WNV *in vivo* [21], but stands in contrast to studies with the alphaviruses Sindbis virus, VEEV, and EEEV [16], [17]–[19] and to recent *in vitro* studies with Dengue virus [20]. Taken together, these previous and current results clearly demonstrate that individual mutations that accumulate during host specialization may result in a range of phenotypes in other hosts, including those which are beneficial, neutral, or deleterious. A recent study with VSV demonstrates that mutations in particular regions of the genome are linked to fitness tradeoffs while other mutations may not be [33]. In mosquitoes, viral growth after passage demonstrated that, with the exception of a single lineage on day 1 p.i., virus titers were either lower or statistically similar to the parental strain (Fig. 4). These results are consistent with previous studies in our laboratory using only SLEV ejected in the salivary secretion of *Cx. pipiens* for passaging [21] and further demonstrate that additional adaptation in terms of replicative ability is not attainable for SLEV in *Cx. pipiens* mosquitoes using these methods. In addition, infectious dose experiments demonstrate that increased infectivity in mosquitoes was also not achieved through sequential passage (table 3).

An important caveat of these experiments is the fact that intrathoracic inoculation rather than infection of midgut cells via bloodfeeding is used for both passage and growth kinetics experiments. It is certainly possible that optimization for infection, replication, and egress from midgut tissue is much different than optimization for infection and replication in exterior tissues, yet achieving adequately high titers for mosquito infection via bloodfeeding in the absence of intermediate amplification is problematic and, therefore, this passage methodology is not feasible. Despite this, the current studies provide more insight than previous *in vitro* studies and demonstrate that suboptimal adaptation in the mosquito is not necessarily a consequence of host cycling for SLEV. These results stand in contrast to previous studies with WNV, which displayed increased infectivity and replicative ability following passage in *Cx. pipiens* [21]. Phenotypic results overall suggest that in spite of cycling high levels of adaptation to both hosts exist for SLEV, yet the result that some potential exists for further adaptation to the vertebrate but not the invertebrate suggests, not surprisingly, that the two are likely not equal partners in virus evolution. *In vitro* studies with VSV suggest that the more persistant phase of the transmission cycle (the invertebrate) may dominate arbovirus evolution [23]. Similarly, *in vivo* studies with WNV demonstrate that the large mutant spectra generated during mosquito infection are maintained during host switching [10]. As with these previous WNV studies, results here suggest that the main source of genetic variation for SLEV is the mosquito (Figs. 2 and 3). Variation among lineages in terms of nucleotide diversity during chicken passage makes determination of representative mutant spectrum size and clear evaluation of selective pressures difficult. Despite this, analysis of change on the amino acid level demonstrates that sequence variation is generally modest in the avian host. Conversely, levels of genetic variation generated in the mosquito were relatively high and generally consistent among lineages (Figs. 2 and 3). Peak SLEV diversity in the mosquito was in fact higher than levels previously measured in similar studies with WNV [9]. These results stand in contrast to previous *in vitro* studies suggesting that the capacity for genetic change of SLEV may be less than that of WNV in mosquitoes, yet in cell culture studies SLEV became highly adapted to the experimental host while here it did not [11].

Differences in genetic diversity generated in the two hosts *in vivo* can likely be attributed to a combination of differing selective pressures and levels of replication. In the mosquito, what remains unclear is how both neutral and selective bottlenecks within the host specifically affect the size and composition of the viral swarm that is ultimately transmitted. Previous studies with WNV demonstrate that virus sequentially passed using salivary secretions is highly homogeneous, suggesting diversity may be purged prior to transmission [21]. The level of consensus change measured among lineages here was variable (table 1), yet the fact that one of two mosquito and two of three chicken lineages accrued just a single base substitution in the entire genome does not support the idea that cycling leads to considerable dampening of fixed sequence change and, therefore, rates of evolution. Consensus amino acid changes that did occur were by in large conservative changes (table 1). One exception was an Arginine (charged polar) to Glycine (nonpolar) change at position 2295 of the NS4B gene. Surprisingly, this change was identified in both SLEV MP20A and SLEV CP20D. It would seem improbable that the same change would be selected for in completely divergent hosts unless it conferred a significant advantage in each. Since there were no measurable gains for SLEV MP20A in mosquitoes it seems likely that this variant already existed in the SLEV P0 mutant swarm prior to passage and that its phenotypic impact is limited.

Taken together, these results indicate that, although there are clearly differing selective pressures in the avian and mosquito portion of the SLEV life cycle, a high level of adaptation exists in both vertebrate and invertebrate hosts and, therefore, significant coadaptation is attainable for arboviruses in spite of host cycling.
