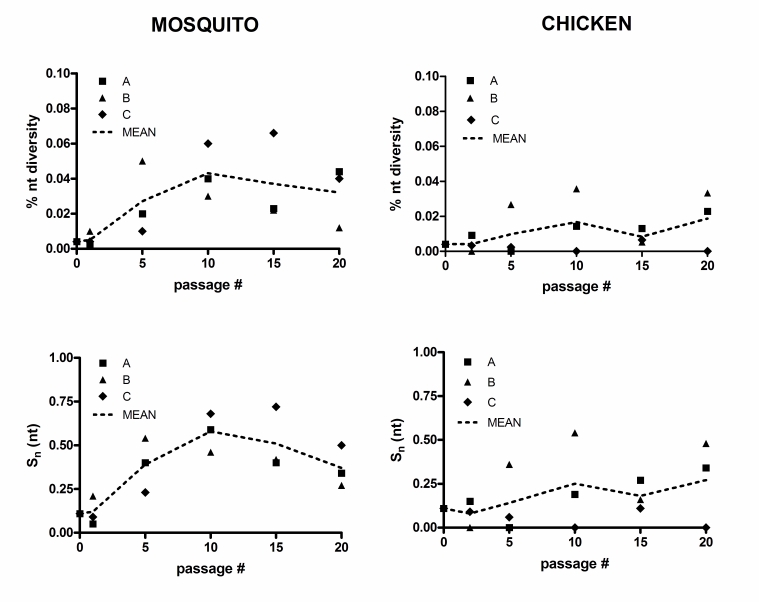# Correction: Experimental Passage of St. Louis Encephalitis Virus *In Vivo* in Mosquitoes and Chickens Reveals Evolutionarily Significant Virus Characteristics

**DOI:** 10.1371/annotation/8e335f18-47c3-4748-b6b7-acca14f94e5c

**Published:** 2010-01-05

**Authors:** Alexander T. Ciota, Yongqing Jia, Anne F. Payne, Greta Jerzak, Lauren J. Davis, David S.Young, Dylan Ehrbar, Laura D. Kramer

In Figure 2, the graphs on the left (mosquito) are duplicates of the graphs on the right (chicken). Please view the corrected Figure 2 here: 

**Figure:**